# Systematic analysis of mRNA 5' coding sequence incompleteness in *Danio rerio*: an automated EST-based approach

**DOI:** 10.1186/1745-6150-2-34

**Published:** 2007-11-27

**Authors:** Flavia Frabetti, Raffaella Casadei, Luca Lenzi, Silvia Canaider, Lorenza Vitale, Federica Facchin, Paolo Carinci, Maria Zannotti, Pierluigi Strippoli

**Affiliations:** 1 Center for Research in Molecular Genetics "Fondazione CARISBO", Department of Histology, Embryology and Applied Biology, University of Bologna, via Belmeloro 8, 40126 Bologna (BO), Italy

## Abstract

**Background:**

All standard methods for cDNA cloning are affected by a potential inability to effectively clone the 5' region of mRNA. The aim of this work was to estimate mRNA open reading frame (ORF) 5' region sequence completeness in the model organism *Danio rerio *(zebrafish).

**Results:**

We implemented a novel automated approach (*5'_ORF_Extender*) that systematically compares available expressed sequence tags (ESTs) with all the zebrafish experimentally determined mRNA sequences, identifies additional sequence stretches at 5' region and scans for the presence of all conditions needed to define a new, extended putative ORF. Our software was able to identify 285 (3.3%) mRNAs with putatively incomplete ORFs at 5' region and, in three example cases selected (*selt1a*, *unc119.2*, *nppa*), the extended coding region at 5' end was cloned by reverse transcription-polymerase chain reaction (RT-PCR).

**Conclusion:**

The implemented method, which could also be useful for the analysis of other genomes, allowed us to describe the relevance of the "5' end mRNA artifact" problem for genomic annotation and functional genomic experiment design in zebrafish.

**Open peer review:**

This article was reviewed by Alexey V. Kochetov (nominated by Mikhail Gelfand), Shamil Sunyaev, and Gáspár Jékely. For the full reviews, please go to the Reviewers' Comments section.

## Background

The amino acid sequence of gene products is routinely deduced from the nucleotide sequence of the relative cloned cDNA, according to rules for recognition of start codon (first-AUG rule, optimal sequence context) and the genetic code [1,2]. The identification of a more complete mRNA 5' end could reveal an additional upstream AUG – in-frame with the previously determined one and in the optimal context – thus extending the predicted amino terminus sequence of the product. We have previously used the term "5' end mRNA artifact" to refer to the incorrect assignment of the first AUG codon in an mRNA sequence, due to the incomplete determination of the mRNA 5' end sequence [3]. The putative translation start based on incomplete mRNA sequence may lead to incorrect prediction of the product amino acid sequence, and to subsequent errors in the experimental cloning and functional assay of the relative cDNA.

*Danio rerio *(zebrafish) is a model organism that has gained popularity for its high suitability for functional genomic experiments. The zebrafish genome project is currently in progress, and about 8,000 mRNAs (out of an estimated 25,000) have been characterized and catalogued in the RefSeq database (available on December 31^st^, 2005). Gene overexpression, gene localization and knock-down protocols are routinely performed on this animal [4], requiring knowledge of the complete open reading frame (ORF) present in the mRNA under study. Moreover, inhibition of expression of specific mRNAs is commonly now achieved by gene knockdown antisense morpholino oligonucleotides (MO) [5], that inhibit translation in a specific manner and are typically targeted against the sequence surrounding the first-AUG codon of the mRNA. Although methods to determine the complete mRNA ORF have been developed, such as 5' cap trapping [6] and cap analysis of gene expression (*CAGE*) [7], they are experimentally intensive and they have not been applied to the zebrafish mRNA on a large scale.

**Figure 1:**
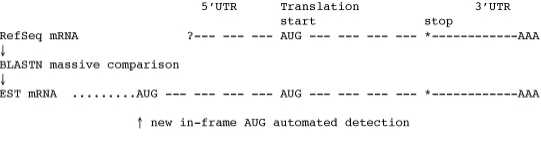
**The mRNA 5' ORF extension pipeline**. Schematic flow of the approach to automated search for mRNA with a putatively incomplete coding sequence: building of a RefSeq mRNA database and of a *Danio rerio *EST database, highthroughput BLAST comparison between the two sequence sets, final elaboration integrating BLAST results and mRNA/EST sequences.

The aim of this study was to implement a novel automated approach(*5'_ORF_Extender *software) able to systematically compare all available expressed sequence tags (ESTs) with all *Danio rerio *experimentally determined mRNA sequences, to identify additional sequence stretches at the mRNA 5' region. The software then scans for the presence of all conditions needed to define a new extended putative ORF: presence of a new first AUG-codon in-frame with the previously described first-AUG, and lack of any in-frame stop between the newly identified and the old first-AUG codons. This required high-throughput sequence analysis performed on a processor cluster and the development of an original relational database able to integrate and analyze data from EST sequences, RefSeq mRNA coding sequences and their relative sequence comparison tabulated results. The software analyzed all the 8,528 *Danio rerio *mRNAs from the RefSeq database (available on December 31^st^, 2005), and was able to identify 285 (3.3%) mRNAs with putatively incomplete ORFs at 5' region (Figure 1). Using RT-PCR and automated sequencing, it was, in fact, found that in three example cases (*selt1a*, *unc119.2*, *nppa*) the coding region at 5' end was incompletely characterized in the originally published descriptions, leading to incorrect predictions for the amino acid sequence of *Danio rerio *selenoprotein T 1a, unc-119 homolog 2 and natriuretic peptide precursor A.

The *5'_ORF_Extender *software allow us to show that 5' end mRNA artifact is not a rare event in the analysis of the zebrafish genome. The implemented method could also be useful for the analysis of other genomes.

## Results

### Database construction and computational analysis

The high-throughput BLAST analysis generated 1,189,412 BLAST hit lines for the 8,528 investigated zebrafish mRNAs compared with the *Danio rerio *EST database. The merged file containing all results was imported into the *5'_ORF_Extender *software.

Following calculations executed by the *5'_ORF_Extender *software, it was possible to obtain candidate extended coding regions at 5' end from 1,346 BLAST hits, using the criteria described in the "Methods" section. This corresponded to 285 distinct zebrafish mRNAs. The mean number of EST sequences that allowed the extension of one mRNA sequence was 4.7, with 141 mRNAs being extended by more than one EST. The data table with complete results for the 1,346 positive BLAST hits (Dr_extended_ORF.txt) is provided as supplemental on-line material along with the software distribution [8].

The mean size of the additional ORF stretch was 67.7 bases, with a standard deviation of 63.7 bases (range: 3–432 bases). Automated results relative to the HC21-homolog zebrafish mRNA test set fully agreed with results of manual analysis.

Due to the process flow of the software, which tests all existent upstream in-frame ATG in the mRNA-extending EST stretch, extended ORFs are detected also in the case of the simultaneous existence of a small ORF upstream of a potential new ATG (upstream ORF or uORF) [9].

### In vitro cloning and sequencing of the mRNA 5' region

Three example genes (*selt1a*, *unc119.2 *and *nppa*) were chosen for the experimental test aimed at verifying the existence of the extended ORF bases in real zebrafish mRNAs, following these criteria: identification of at least two EST sequences showing the extended ORF, presence of at least one published report about the corresponding mRNA, and predicted product extension of at least 30 amino acids.

A cDNA corresponding to the predicted additional coding region was obtained for each of the investigated genes. The nucleotide sequences were deposited in the GenBank database under accession numbers: DQ650637 for *unc119.2 *gene, DQ660904 for *selt1a *gene, DQ787202 for *nppa *gene.

The extended coding sequences for *selt1a*, *unc119.2 *and *nppa *were analyzed using the BLAST program to compare them with known nucleotide and amino acid sequences deposited at NCBI databases. The nucleotide and amino acid analysis data are summarized in Table 1.

**Table 1:** Exemplificative zebrafish genes with extended cDNA 5' region and deduced protein.

*Gene *(RefSeq#)	Error type^a^	GenBank EST# Zebrafish^b^	Genomic clone #	Product length new/old (no. of new amino acids)	Kozak sequence old (top)/new (bottom). Consense^c^: GCCRCC**ATG**G	GenBank EST# Non-zebrafish
*selt1a *(NM_178290)	ND	CN505709	-	196/163 (33, +20%)	ATGAAG**ATG**C	-
		CK681469			CTGATC**ATG**G	
		CN018643^d^				
*unc119.2 *(NM_205713)	1	CN505408	BX465229	264/206 (58, +28%)	GAGGCC**ATG**A	pp DT261717^e^
		CK363344	BX005137		CGGATA**ATG**A	pp DT134309
		BI710727^d^				pp DT116366
						pp DT263287
*nppa *(NM_198800)	1, 2	CN176149	BX323876	139/106 (33, +31%)	AGCAAC**ATG**G	-
		CN180261			TCAGAG**ATG**G	
		CO929886^d^				

^a ^(1) extended exon 1; (2) new exon; ND: not determined owing to unavailability of genomic sequence.

^b ^GenBank sequences matching extended coding sequence from the new start codon in EST (Expressed Sequence Tag) division.

^c ^The two most conserved positions (Kozak, 1999; Kozak, 2002) are underlined; start codon, in bold font.

^d ^Only three representative sequences are listed, out of a total of 24 for *selt1a*, 4 for *unc119*.2, and 26 for *nppa*, showing consistent coding sequence extension (see Table online).

^e ^pp = *Pimephales promelas *fish.

**Figure 2:**
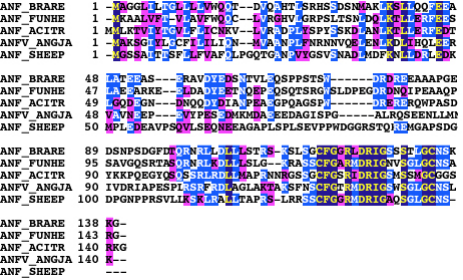
**ClustalW alignment of atrial natriuretic peptide (ANP) sequences from different species**. Sequence for zebrafish (BRARE) ANP is derived from the extended *nppa *cDNA sequence we present here; methionine in position 34 is the previously described first amino acid. Other ANP amino acid sequences are from: white sturgeon (*Acipenser transmontanus*, ACITR), killifish or mummichog (*Fundulus heteroclitus*, FUNHE), Japanese eel (*Anguilla japonica*, ANGJA), sheep (*Ovis aries*, SHEEP).

The newly found translational start codons all show a better consensus with the Kozak sequence. The amino acid sequences predicted at the amino terminus in these three cases did not show new known functional domains through database searches. The coding nature of these upstream bases was further confirmed by phylogenetic comparison at amino acid level in the case of *nppa *product (Figure 2).

## Discussion

The problem of completeness of mRNAs in order to define at least one complete ORF is a well known topic in molecular biology and genomics [10]. Anecdotal evidence of the presence of a further extension of mRNA 5' end sequence, with consequent correction of the previously accepted predicted product, was randomly found for single genes. In addition, several studies have estimated that the percentage of possible mRNA coding sequence incompleteness in whole genome may be in the range of 4–5% [3,11], and the analysis of 90,000 human 5'-end-enriched cDNA clones has indicated that ~380 known gene models could be extended at their 5'-end [12]. Relevant consequences may arise from the incomplete prediction of the gene products and they have previously been described [3].

We wondered if errors consistent with the 5' end artifact might be present in other genomes. We then decided to systematically analyze the extent of completeness of the whole set of known mRNAs described in zebrafish, for the relevance in the design of post-genomic experiments, for the recent availability of most mRNA and genome zebrafish sequences, and for the relatively small size of the EST species-specific database section (about 10% of the human section), which could represent a suitable starting point for the feasibility of the development of new tools.

Our new systematic and automated approach to the 5' end mRNA artifact makes use of the first type of software able to automatically integrate large volumes of mRNA sequence data, EST sequence data, and sequence comparison data with respect to existent and possible initiation codons in order to generate EST-driven models of 5' ORF extension on a genomic scale. In particular, it integrates EST analysis aimed at extending the current mRNA sequence with the known mRNA translation frame, thereby differing from other methods which do not incorporate prediction of the ORF extension [e.g. [13]]. This software allowed us to improve on our first manual sequence analysis performed in a subset of zebrafish genes with homology to HC21 human loci (data not shown), and to quickly obtain a useful list of putative incomplete ORFs in zebrafish. The software can run on both Mac OS X and Windows operated personal computers and it is available for free for anyone to use and modify. A limit of the software is that it is focused only on the plus/plus alignments (i.e. with sequences in the same orientation) in the generation of the extended mRNA models. However, most ESTs are directionally cloned cDNAs, and it is likely that this limit only slightly lowers the sensitivity of the program. This implies that the actual proportion of mRNAs with incomplete ORFs may be even larger than estimated here, also considering that sensitivity of the method depends on the EST repertoire available at the moment of the analysis.

Genomic browsers continuously scan deposited sequences and try to build mRNA models. They employ different methods and algorithms, however they appear not to systematically address the item we discuss. Following comparison of our three example confirmed mRNAs (*nppa*, *selt1a *and *unc119.2*), with data available in NCBI Map Viewer, UCSC Genome Browser and EBI Ensembl, we found that none of these present the extended models we have determined and validated. A prediction consistent with our results was found only for *unc119.2 *and only in NCBI (predicted sequence XM_697866). We conclude that our approach is not included in the pipeline of these reference map browsers and it is the first able to generate, on a large scale, extended models of the actual 5' ORF.

A total of 285 genes, out of the 8,528 zebrafish gene-set, have been recognized as candidates for extension. It is strongly supposed that mRNAs for which more than one EST was found leading to the same prediction, possess a longer ORF than that described. Three representative mRNAs were extended, demonstrating a new coding tract and thus identifying an open reading frame with extra amino acids to those reported by the Authors who originally described these genes: *selt1a *(supported by 24 consistent ESTs), *unc119.2 *(supported by 4 consistent ESTs) and *nppa *(supported by 26 consistent ESTs). In these cases, both of the following conditions occurred: an extension of described exon 1 predicted new coding codons upstream of the known AUG; and a novel AUG was present upstream of these codons, in-frame with the previously described AUG and without any intervening stop codon. Following the rules of translation initiation [1,2], the actual coding sequence should be considered as that included between the novel "first-AUG" and the known stop. However, our identification of the most upstream currently definable AUG start codon does not exclude that a downstream AUG codon may also be used by the ribosome, due to the phenomenon of alternative translation [14,15]. It should also be noted that this type of analysis cannot formally exclude that the extended ORF may derive from alternative transcription starting site and/or splicing of the investigated locus. The coding nature of these upstream bases is further confirmed by phylogenetic comparison at amino acid level with several species in the case of *nppa *product (Figure 2) and with *T. nigroviridis *in the case of *unc119.2*, making it unlikely that extension is due to alternative transcription of the gene. Therefore the predicted product for these three zebrafish genes should be redefined for functional studies.

Many extended mRNAs in our list derive from the large-scale cDNA sequencing project, but for a number of these mRNAs, publication of the sequence as well as molecular biology experiments based on this sequence were reported. Regarding the cases that we have studied in detail, *selt1a *mRNA incompleteness was without consequences in the article by Thisse et al. [16]. In this article, *selt1a *cDNA clones are used as *in situ *hybridization probes, which are sufficiently specific, even if partial. Regarding *unc119.2*, Manning et al. [17] described a predicted product (named zfunc119b) which appeared to be the shortest among the UNC-119 highly conserved metazoan family of proteins they reconstructed, but it actually lacks the additional coding sequence at 5' we describe here. This led to MO knockdown experiment design referring to the putative, incorrect translation start codon. Although it is unlikely that this may have significantly affected the resulting "curly tail down" phenotype (joined with a constellation of neuronal defects similar to those seen in *C. elegans *unc-119 mutants), MO canonical method requires targeting the first actual translational start codon. Finally, Berdougo et al. [18] described the *nppa *mRNA sequence in zebrafish, encoding natriuretic peptide, an important hormone involved in the control of body fluid volume, produced by the heart. These Authors assembled a consensus cDNA sequence from the ESTs available at the time, with incomplete 5' region. Although this information was only used to design hybridization probes for the study of mRNA expression, the predicted *nppa *product lacks 33 amino acids at the amino terminus that are conserved among different fish and also in some mammals (Figure 2).

## Conclusion

This study has presented a novel high-throughput strategy to search for mRNA sequence incompleteness by generating new mRNA models via EST and RefSeq database integrated analysis. Our findings in the *Danio rerio *underline the need for accurate analysis of mRNA and protein sequences when planning functional experiments in this model organism, with regard to a significant presence of the 5' end mRNA artifact in the reported mRNA sequences for this species. Moreover, the implemented method could also be useful for the analysis of other genomes.

## Methods

### Database construction and computational analysis

The *5'_ORF_Extender *software was developed to parse RefSeq and EST sequence databases and to make calculations on the sequences, following the import of BLAST results. It was developed using FileMaker Pro 8 Advanced (FileMaker, Santa Clara, CA) database management system for both Windows (2000 or above) and Macintosh (10.3.9 or above) operating systems, and it is available as a stand-alone software including the FileMaker runtime. The software is freely available, with a user guide included [8]. Any modification of the software is also allowed, but in this case the user must purchase the FileMaker application. The software was used on a Power Mac G5 dual processor computer (Apple, Cupertino, CA).

Editing of large text files is required in several steps and we best performed it using common Unix-like utilities included in Mac OS X ("tr", "awk", "cat"), available on any Unix/Linux system. The zebrafish RefSeq flat file, containing all known reference mRNA sequences for *Danio rerio*, was downloaded from the NCBI ftp site on December 31^st^, 2005. A Mac OS X script was developed and run to format the RefSeq entries for import into the appropriate *5'_ORF_Extender *database table (see the software user guide). The RefSeq table includes calculations aimed at extracting several key features of the GenBank RefSeq entry: in particular, LOCUS and 5' UTR length.

The zebrafish EST entries, including all known ESTs for *Danio rerio *in the dbEST database, were downloaded from the NCBI website via Entrez on December 31^st^, 2005. A script was run as described above to format the EST entries for import into a local EST database template. The software includes calculations aimed at extracting the GenBank EST sequence length and the EST entry LOCUS name, in order to establish relationships useful for the automated search of ORF extension at mRNA 5' end (see below).

Each *Danio rerio *mRNA catalogued in the RefSeq database (total: 8,528 entries, excluding "XM_" type, non-reviewed predicted entries) was compared using BLAST software with all the *Danio rerio *ESTs present in the EST database. To automate this process, we ran BLAST 2.2.11 software on the IBM CLX/1024 cluster at CINECA – High Performance System Center, with expect = 2e-12 (see the software user guide for details). All comparison data refer to sequence databases available on December 31^st^, 2005 as described above. The BLAST result data files were imported into the appropriate fields of a FileMaker Pro 8 database template developed for this research (*5'_ORF_Extender*). During this import process, relationships were created by the software using the respective accession number as the key field. These relationships point to the appropriate fields in either RefSeq or EST databases, created as described above. This allowed us (within each record corresponding to a blast hit) to make some values available which were not present in the BLAST results table, such as the sequence and length in bp of 5' UTR RefSeq mRNA "query" sequence, and the sequence and length in bp of EST "subject" sequence entry.

A script was then written and executed so as to subsequently: extract the EST sequence stretch upstream of the matched RefSeq mRNA first base in all BLAST hits showing a 5' extension of the EST sequence with respect to the known RefSeq sequence; search in this EST stretch for the most upstream existent ATG in-frame with the described one in the RefSeq mRNA sequence entry; calculate the new putative extended coding region (by merging the EST extended stretch starting from the new ATG with the 5' UTR of the RefSeq mRNA sequence); confirm the coding potential of this new extended sequence by excluding the presence of any in-frame stop codon within it. The software screens RefSeq mRNAs for the presence of an in-frame stop codon upstream of the described initiation codon in the mRNA sequence itself, because this indicates that the recorded 5' UTR sequence cannot be part of a longer continuous coding sequence. Consequently, this subset of mRNAs is analyzed no further.

The script then analyzes only BLAST alignments matching these criteria: mRNA sequence query "start" position is "1" and EST subject sequence "start" position is greater than "1", in order to be sure that only EST sequences containing additional upstream nucleotides with respect to the known most upstream mRNA sequence are analyzed; EST subject sequence start position is not greater than the EST subject sequence "end" position, in order to focus the analysis on the plus/plus alignments (i.e. with sequences in the same orientation) in the generation of the extended mRNA models.

To avoid artifacts due to poor alignments between the mRNA and the EST sequences, only alignments with a percentage of nucleotide identity equal to or greater than 97% and with a length of the EST sequence aligned with the mRNA greater than 49% of the total EST length were selected. These parameters are stringent but they may be modified by the user if desired. Lowering these values may allow further identification of extended ORFs when the mRNA sequence only partly aligns with the EST sequence, due to the existence of ESTs longer than the whole respective mRNA or to alternative splicing outside the aligned region, at the risk of possible retrieval of false positive ORF extensions.

### In vitro cloning and sequencing of the mRNA 5' region

An *in vitro *cloning approach was devised to confirm the sequence analysis predictions. RT-PCR was performed, mainly based on amplification of a more complete AUG-containing first exon, extended from the new putatively defined 5' UTR and one downstream exon, in order to be sure that amplified cDNA derives from mRNA.

Total RNA was extracted from ~60 zebrafish embryos at shield stage, and from the whole body of a 4-month adult, using the Chomczynski and Sacchi method [19]. Fish and embryos were maintained in our own facility according to standard procedures [20].

For RT-PCR, 1 μg of total RNA was reverse transcribed and amplified using the appropriate primer pairs. PCR amplification and sequencing techniques were performed in standard conditions [3].

The extended coding sequences for *selt1a*, *unc119.2 *and *nppa *were analyzed using the BLAST program to compare them with known nucleotide and amino acid sequences deposited at NCBI databases. The predicted extended amino acid sequences for the 3 genes were searched for in several domain databases, such as SMART [21] and CDD [22], to identify new domains that were not present in the described gene product. Alignment of the natriuretic peptide sequences from different species was performed using ClustalW software (version 1.8).

## Abbreviations

5' UTR, 5' untranslated region; BLAST, Basic Local Alignment Search Tool; BLASTN, Blast nucleotide-nucleotide; bp, base pairs; CDD, Conserved Domain Database; cDNA, DNA complementary to RNA; dbEST, EST database; dNTP, deoxyribonucleoside triphosphate; EST, expressed sequence tag; HC21, human chromosome 21; mRNA, messenger RNA; MO, morpholino oligonucleotides; PCR, polymerase chain reaction; RT-PCR, reverse transcription PCR; SMART, Simple Modular Architecture Research Tool.

## Competing interests

The author(s) declare that they have no competing interests.

## Authors' contributions

All authors participated in the study design and in the performance of the experimental procedures; they all drafted the manuscript and approved the final version.

## Reviewers' comments

### Reviewer's report 1

*Dr. Alexey V. Kochetov (nominated by Mikhail Gelfand), Institute of Cytology and Genetics, Novosibirsk, Russia*.

The manuscript concerns an interesting and important problem of prediction of eukaryotic mRNA 5'-terminal part and translation start site. The authors prepared a tool for automatic comparison of EST and cDNA data sets. Additional information from EST clones allows revealing 5'-ending incomplete cDNAs and correcting them. Some examples of predicted 5'-end extended *Danio rerio *cDNAs were selected and verified experimentally, which proved usefulness of the method.

In my opinion, the problem of correct prediction of both 5'-end of mRNA and translation start sites is far from being solved. Accuracy and sensitivity of available computational tools are limited (e.g., Nadershahi et al. Comparison of computational methods for identifying translation initiation sites in EST data. BMC Bionformatics. 2004. 5:14). Additional analysis of full-size mRNAs is of importance to reveal new mRNA and protein variants (e.g., Casadei et al. mRNA 5'-region sequence incompleteness: a potential source of systematic errors in translation initiation codon assignment in human mRNAs. Gene 2003 32 185–193; Porcel et al. Numerous novel annotations of the human genome sequence supported by a 5'-end-enriched cDNA collection. Genome Res. 2004. 14. 463–471) Thus, new information and software resources in this field are valuable.

1) EST data are very frequently used to map the gene structure. As I understand, the approach of Frabetti et al. is likely to be characterized by less strict limitations on the EST usage that allowed getting more information in comparison with other investigations. For example, Kitagawa et al. (Bioinformatics. 2005. 21. 1758–1763) have also used EST multiple alignment to map mRNA 5'-end (transcription start sites). They removed 10% of 5'-farthest ESTs as outliers because "...they did not need a large quantity of TSS (transcription start site) datasets but rather accurate ones". Probably, application of more strict limitations increases accuracy but decreases sensitivity. Frabetti et al. proved the efficiency of applied criteria experimentally. However, I would like to know their opinion. Probably, this question may also be discussed in more detail in the manuscript to address the difference between the Authors approach and other available tools. To this point: some comparative (brief) review of different prediction methods and tools might increase the manuscript quality considerably. It would be also interesting to know: how many zebrafish gene models corresponding to these 285 potentially 5'-extended mRNAs (and available in databanks) coincide with the mRNA model predicted by the Authors?

**Author's response: ***The aim of this work is not to predict the mRNA 5'-end and translation star site, but to exploit the currently available EST dataset to improve the present knowledge about the most complete ORF which can be assigned to a known mRNA. In this respect, our tool does not start from EST sequences as in the case of the tools compared in Nadershahi et al., which work independently of the known reference translation start site, but on the contrary it examines known mRNAs to verify the possibility of building more extended models at 5'-end that include an extension of the currently accepted ORF. Similarly, the approach described in rice by Kitagawa et al., is aimed to first create EST clusters, then to identify ORFs, rather than check completeness of known ORFs. In addition, these Authors do not release a package for general use of their method. The work by Kitagawa et al., 2005, has now been briefly discussed and cited in the third paragraph of the Discussion*.

*To our knowledge, available software to compute mRNA ORF models with the best possible ORF 5'-end is included in the pipelines of the major genome browsers (NCBI MapViewer, UCSC Genome Browser, Ensembl). We have already shown in the article that our predicted and experimentally validated extended models were not computed by these reference map browsers. As the reviewer suggests, we believe that it is due to differences in the approaches used. While these programs use complex computations including statistical analysis to build their models, our software is more sensitive in the specific task of mechanically finding any EST which may allow the extension of a known mRNA ORF, considering its already known translation frame. Searching for potentially 5'-extended mRNAs into NCBI sequences, including both finished and predicted sequences, only 29 out of the 285 are available to date (August 2007). In addition, in several cases the extension was made available only after we had conducted our analysis, due to the release of new finished (non-EST) sequences in the databases, as in the case of the three mRNAs we have experimentally validated in this work and we ourselves have submitted to GenBank. A more detailed discussion of this item would require a systematic comparison of the details of the pipelines used by the genome browser to build their gene models, which is beyond the aim of this work. Moreover, the originality of our software is also in allowing simple large-scale analysis of BLAST results in a database framework, e.g. producing the subset of all mRNAs extended by the analysis for a whole transcriptome of an organism*.

*The work by Porcel et al., 2004, has been briefly discussed and cited in the first paragraph of the Discussion*.

2) Many eukaryotic genes produce several mRNA variants with different 5'-ends because of the usage of alternative promoters and alternative splicing. It was recently evaluated that in mouse transcriptome there were about 1.32 5' start sites for each 3'-end (The FANTOM consortium et al. The transcriptional landscape of the mammalian genome. Science. 2005. 309. 1559–1563). Actually, comparative analysis of available EST data can be used to reveal mRNA 5'-end heterogeneity. For example, multiple alignment of 5'-EST sequences allowed revealing numerous multiple transcription start sites and alternative first exones in rice and mouse (Kitagawa et al. Computational analysis suggests that alternative first exones are involved in tissue-specific transcription in rice. Bioinformatics. 2005. 21. 1758–1763). However, Frabetti et al. considered only the situation of 5'-end incomplete mRNAs rather than an opportunity of synthesis of alternative mRNA forms producing different protein isoforms. What is the reason for such a limitation? May some "artifacts" be just additionally produced alternative forms?

**Author's response: ***Our research simply aimed to identify cDNA ORF sequences longer than those previously suspected. The systematic analysis of transcription start sites and/or alternative splicing would obviously require the incorporation of the whole-genome sequence, provided that it is available for a given organism, and the development of heavy computations to build mRNA models accounting for alternative transcription and/or splicing. This is beyond the aim of this work, and is also probably beyond the capability of the FileMaker Pro software running on the present personal computers*.

*Regarding the possibility that an mRNA model extended by our approach could represent an isoform due to alternative transcription and/or splicing, we cannot formally exclude this possibility. As in the case of any other computer prediction, further investigation is required, in silico but especially in vitro, for a fine characterization of the putative model. However, we would underline that our program adds new sequence information starting exactly from the first base of the 5' end of a known mRNA form, revealing coding bases that were previously considered to be untranslated. Actually, if the more complete sequence had been cloned in the original work, the Authors would certainly have registered the upstream in-frame AUG as the start codon. We have added a brief discussion of this item in the fifth paragraph of the Discussion section*.

3) I also have one comment concerning the prediction of translation start site (TSS). The Authors used Kozak's rules to predict the position of start AUG codon (context and 5'-proximal position). This is a common method and it may be used. However, I would like to note that there is a discrepancy between the experimental data and bioinformatics approaches. According to the scanning model, 40S ribosomal subunits are recruited to the 5'-terminal cap structure, scan in a 5'- to-3' direction, and can initiate translation at the first AUG they encounter (Kozak. Regulation of translation *via *mRNA structure in prokaryotes and eukaryotes. Gene. 2005. 361. 13–37.). If context of 5'-proximal AUG codon is suboptimal, some 40S ribosomal subunits recognize it as a translational start site, but other will miss it, continue scanning in 3' direction, and initiate translation at downstream AUG (leaky scanning). The usage of alternative translation start sites is quite possible (some examples were demonstrated experimentally). This opinion was also recently supported by analysis of interdependency between the TSS context and the presence of AUG codons at the CDS beginning (Kochetov. AUG codons at the beginning of protein coding sequences are frequent in eukaryotic mRNAs with a suboptimal start codon context. Bioinformatics. 2005 21. 837–840; Kochetov et al. The role of alternative translation start sites in generation of human protein diversity. Mol. Genet. Genomics. 2005. 273. 491–496). Thus, suboptimal AUG context does not necessarily mean that this AUG is not used as a start site. This is not a problem of this particular manuscript: currently most gene prediction programs do not take into account alternative translation start sites. This comment does not need a reply.

**Author's response: ***We agree that a brief comment about alternative translation is appropriate, and we have added a sentence and two references in the fifth paragraph of the Discussion about this item*.

Other comments:

Actually, I am not sure that the Authors need to attract so much attention to the importance of problem of cDNA 5'-end incompleteness: it is quite clear that many cDNAs are incomplete. It may be more reasonable to concentrate attention on the methods (and software) to solve this well known problem rather than emphasize the problem importance itself.

English language should be improved.

**Author's response: ***In the Introduction and Discussion we have concentrated our attention on the method to solve the problem rather than on the known importance of the problem, as suggested. The English has been revised*.

### Reviewer's report 2

*Dr. Shamil Sunyaev, Harvard Medical School, Boston (MA)*

This manuscript contributes to the problem of accurate annotation of sequenced genomes. The authors argue that inaccurate determination of transcription starts may lead to false annotation of translation starts. Using analysis of EST sequences they identified translation start sites located 5' of the annotated sites for a few percent of zebrafish genes. They experimentally prove the existence of these larger transcripts for three zebrafish genes.

I have a few comments on this version of the manuscript:

1) Eukaryotic transcription start sites are known to be frequently wobbly and sometimes there are alternative sites which may be tissue or developmental stage specific. Is it possible that the authors detect alternative starting sites rather than 5' artifacts? The manuscript would greatly benefit from a discussion of this point.

**Author's response: ***The same point was also raised by Reviewer 1, please see response to Reviewer 1, point 2 for what alternative transcription is concerned*.

2) The authors use 97% sequence identity threshold for EST alignments (it seems without constraining alignment length). Is this sufficient to avoid aligning EST belonging to paralogous genes? In other words, is it possible that ESTs used by the authors correspond to transcripts of different genes?

**Author's response: ***As we note in the Methods section, 97% of the sequence identity parameter is stringent but it may be modified by the user if desired. It was chosen considering the known recurrence of sequencing errors in the EST entries. Actually, we also point out that we allow the option of a constraint of alignment length and that we used 49% of the total EST length, empirically determined*.

*Regarding the possibility of detecting ESTs related to paralogous genes in the same query, we first observe that coding-gene paralogy within a species may assume mean values different from those found in a different species. However, in general, the percentage of identity between paralogous genes in a gene family is well below 90%. For example, in the case of the organism we study in this work (zebrafish), a simple analysis of two classic gene families known to have a very high grade of conservation among their members (homeobox and histone gene families) reveals that, at nucleotide level, values of sequence identity for each pair comparison were up to 80% (e.g., typically in the range of 71–80% in the case of homeobox)*.

3) It is unclear why the software is currently limited to plus/plus alignments and why so many manual steps are needed. Are there any serious hurdles for development of a fully functioning software?

**Author's response: ***We have limited the software to plus/plus alignments, for two reasons: first, it is implemented in a general-purpose database software for common personal computers, and computations to reverse the sequence in order to resolve plus/minus alignments would currently be very heavy and slow in this situation. However, we explicitly chose this graphical interface to make the software easier for biologists with limited IT skills to use. In addition, as we point out in the Discussion, most ESTs are directionally cloned cDNAs, in particular those deriving from more recently obtained libraries that are also, due to experimental improvements, those with a better representation of the cDNA 5' end. In the case of directional cloning, the reverse sequence of the EST is not expected to add data about the 5' end*.

*We agree that some manual work is at present necessary to make the software run. However, due to the potentially long time required to import data, we prefer to keep the execution of each main step separate. In addition, it is difficult to incorporate the procedure in a single command, because it exploits different programs and web sites to be used on a personal computer. The use of AppleScript could be useful on Macs in order to combine the different required instructions, however many programs are not fully scriptable and in any case this system script language is not easily available in Windows OS. We look forward to improvements in personal computer OS scripting languages and software to make the procedure more compact in future versions of the software. Anyway, we have now added an example test to the software distribution to make the instructions clearer*.

4) I would recommend omitting straightforward computational details of the procedure from the manuscript.

**Author's response: ***The details about the procedure have been removed and they remain available in the Guide provided along with the software*.

### Reviewer's report 3

*Gáspár Jékely, European Molecular Biology Laboratory, Heidelberg, Germany*

This paper describes a novel software tool that searches for mRNA 5' sequence incompleteness at a large scale and the application of this tool for zebrafish mRNAs. Such an EST-based approach is important in order to improve mRNA completeness and the prediction of the correct translation start site in genome databases. The method described adequately addresses the problem of mRNA incompleteness in databases.

The description of the problem, the methods, and the three experimentally validated examples is somewhat lengthy and redundant. The methods section is also unnecessarily detailed and should be shortened. For example the section starting with "The Danio rerio EST subset was also downloaded from Entrez in GenBank format" contains trivial details about data download, concatenation, database formatting and local blasting. The methods section should focus on describing how the software developed by the Authors works.

**Author's response: ***The description of the problem, of the methods and of the examples has been revised as suggested. The details about the software have been removed and they remain available in the Guide provided with the software. The English has been revised*.
